# Treatment and disposal practices of pharmaceutical effluent containing potential antibiotic residues in two states in India and perceptions of various stakeholders on contribution of pharmaceutical effluent to antimicrobial resistance: a qualitative study

**DOI:** 10.1186/s40545-023-00562-z

**Published:** 2023-05-01

**Authors:** Anita Kotwani, Ajita Kapur, Mihir Chauhan, Sumanth Gandra

**Affiliations:** 1 Department of Pharmacology, Vallabhbhai Patel Chest Institute, University of Delhi, 110007 Delhi, India; 2 Division of Infectious Diseases, Barnes-Jewish Hospital, Washington University School of Medicine, Box 8051, 4523 Clayton Ave., Campus, Saint Louis, MO 63110 USA

## Abstract

**Background:**

Antimicrobial resistance (AMR) is a looming pandemic, demanding prompt actions to avert catastrophic consequences. Effluents from pharmaceutical industries containing antimicrobial residues could serve as one of the entry points of these drugs to the environment. This qualitative study explores the treatment and disposal practices of pharmaceutical effluent (PE) containing potential antibiotic residues (ARs) by interviewing major stakeholders. In addition, we assessed their knowledge and perception on contribution of PE to AMR.

**Methods:**

The study was conducted in the two Indian states, Haryana and Telangana and at the federal level. Data was collected by semi-structured in-depth interviews of 29 participants from 17 stakeholders/organizations viz. Central Pollution Control Board (CPCB), State Pollution Control Boards (SPCBs) of Telangana and Haryana, civic body, pharmaceutical manufacturers, pharmaceutical associations and civil society. Data was analyzed using thematic analysis.

**Results:**

The effluent treatment and disposal practices varied with the multinational companies (MNCs) having advanced technologies whereas the small and medium-scale pharmaceutical companies (SMPCs) having effluent treatment plants as per the regulations but often under-utilized. The presence of ARs in the PE was considered inconsequential by SPCBs and SMPCs and majority of stakeholders imputed other causes as major contributors to AMR. However, the MNCs were well aware of the contribution of PE to AMR and CPCB also considered ARs as direct source of AMR. The central regulators as well as MNCs expressed concerns regarding the current regulations lacking maximum ARs in the PE.

**Conclusion:**

Setting up regulatory standards for maximum ARs in PE, their implementation and monitoring is an urgent need to curb environmental contribution of ARs to AMR. The findings of our study will help in systematic approach in policy making, awareness programs and capacity-building in dealing with the ARs in PE to combat AMR.

## Background

Antimicrobial resistance (AMR) is a looming pandemic, demanding prompt actions to avert catastrophic consequences. Misuse of antimicrobials in human, animal and environmental sectors is one of the major driving factors for AMR. Environment can act as repository for AMR by transfer of resistance genes from environmental microbes to pathogenic species and selection of resistant organisms in the presence of antimicrobial contamination of soil and waterbodies [1]. Humans and animals could then be exposed to these resistant microbial strains even without direct consumption of antimicrobials. Effluents from pharmaceutical industries containing antimicrobial residues could serve as one of the entry points of these drugs to the environment. Studies from both developed and developing countries including India have documented the presence of high levels of antibiotic residues (ARs) in the pharmaceutical effluents (PE) [2–5]. Currently, there are no clear guidelines on effluent treatment for antibiotics or permissible levels of ARs in the effluents by any country or international agency [6].

Globally, the pharmaceutical industry of India is 3rd largest and 14th largest in terms of volume and value respectively [7]. It is the leading provider for the generic drugs in the world. The Indian pharmaceutical market is involved in each stage of the production process including active pharmaceutical ingredient (API); pharmaceutical formulation intermediates (PFI); and finished dose products (FDP) [8]. There are more than 3000 pharmaceutical companies in India; which are classified as small-scale, medium-scale, large-scale, and multinational companies [9]. The wastewater effluents from the antibiotic manufacturing units contain a substantial amount of antibiotics, leading to contamination of rivers and lakes [10, 11]. In India, Central Pollution Control Board (CPCB) is the national statutory organization entrusted with providing guidance and technical support for establishing effluent standards for pharmaceutical industry waste which are taken up by all State Pollution Control Boards (SPCBs). The current standards include monitoring of pH, biochemical oxygen demand (BOD), chemical oxygen demand (COD), oil and grease, total suspended solids (TDS), and bioassay test but ARs are not included [12].

It is important to understand the current PE treatment and disposal practices by pharmaceutical industries as this would assist in policy making and developing awareness programs to successfully alleviate the contribution of PE to AMR. This qualitative study explores the treatment and disposal practices of PE containing potential ARs by conducting in-depth interviews of important stakeholders such as representatives from pharmaceutical manufacturing units, regulatory authorities at national and state level. In addition, we assessed their knowledge and perception of the contribution of PE to AMR.

## Methods

This was a qualitative study which involved collection of data by semi-structured in-depth interviews from major stakeholders involved in regulation, manufacture, treatment and disposal of PE as well as from a civil society. The stakeholders were identified using system design & network-mapping. Regulators (CPCB and SPCBs), civic body, pharmaceutical manufacturers, pharmaceutical associations and civil society were identified as major stakeholders. The two states chosen were one from the North India, Haryana (H) and the other from the South India, Telangana (T) as these two states are pharmaceutical hubs in North and South of India [11]. Participants were selected in the study based on purposive sampling and included as per their willingness to participate. Overall, 29 participants from 17 different stakeholders were interviewed for this study.

### Study respondents

The details of the study respondents including mode and duration of interview/discussion are given in Table 1.

**Table 1:** Details of the in-depth discussion with various stakeholders that participated in the study

Stakeholder	Region	Organization	Respondent	Mode of interview	Duration of interview (min)
Regulatory bodies	Centre	CPCB	Chairman, Additional Director	Online	20
	Haryana	SPCB	Scientific engineer	Face to face interview	40
	Telangana	SPCB	Chief environmental engineer	Face to face interview	60
Civic body	Hyderabad	Municipal Cooperation	Chief general manager, Assistant of Chief general manager	Face to face interview	60, 15
Pharmaceutical associations	Haryana	Pharmaceutical manufacturer association of a city	President, Secretary, Member	Face to face interview	70
	Hyderabad	Bulk drug manufacturer association	Executive director	Face to face interview	80
	Mumbai	Organization of Pharmaceutical Producer of India	Director General and Senior Director of Communication and Patient Advocacy	Online	60
	Mumbai	Indian Pharmaceutical Alliance	Secretary general and Member	Online	60
Antibiotic manufacturing pharmaceutical companies	Haryana	Small-scale Indian formulation company (manufacturing injectable beta and non-beta lactam)	Owner & quality control officer	Face to face interview	70
		Medium-scale Indian formulation company (manufacturing oral non-beta lactam antibiotics)	Promoter	Face to face interview	75
		Large-scale Indian API manufacturing company	Technical director	Face to face interview	80
		Large-scale Indian API manufacturing company	Managing director	Face to face interview	70
		Multinational API manufacturing company	Technical operations manager, co-operate sustainability officer, quality control officer and waste management officer	Face to face interview	80
	Telangana	Medium-scale Indian formulation company	Head of Formulation/Deputy general manager	Face to face interview	70
		Multinational API & formulation company	Associate vice-president	Face to face interview	75
		Multinational API manufacturing company	Plant manager, chemical engineer and a safety respondent	Face to face interview	80
Civil society	Delhi	Centre for Science & Environment	Program Director	Face to face interview	70

*CPCB* Central Pollution Control Board; *SPCB* State Pollution Control Board; *API* Active Pharmaceutical Ingredient

### Data collection

Semi-structured in-depth interviews of the respondents were conducted following verbal witness consent to collect data on knowledge, attitude and practices on the treatment and disposal practices of PE, contribution of PE to AMR, as well as regulations regarding the same. We conducted face-to-face in-depth interviews with most of the stakeholders, however, due to COVID-19 lockdown, the in-depth interviews for some of the stakeholders were conducted through Google Meet (Table 1) using the Interview Guide. For new issues that emerged during the interview, additional probing questions were asked without overtly leading the respondent to avoid bias. Data was collected by the research team between October 2019 to July 2020 in the language preferred by the respondents (English and Hindi) and audio-recorded with prior consent. The participation was voluntary, and anonymity was maintained.

### Data analysis

Qualitative data was analyzed using thematic analysis [13]. The interviews were transcribed in MS word from the audio recording and translated from the local Hindi language (wherever required) to English. Further data analysis was done manually in MS Word. As initial step to aid familiarization, the research team read and re-read each transcript, before carefully coding each transcript line-by-line. The data was coded in MS Word and Excel sheets were used by two research fellows independently and subsequent reviews were done for thematic analysis. The coding process was both inductive and deductive, as some themes were derived inductively, while others deducted from the data [13]. Researchers deliberated on any diverging issues in coding and arrived at consensus with the principal investigator (AK). All major themes and sub-themes were agreed with discussion and consensus of the entire team.

## Results

Seventeen stakeholders were interviewed and four themes with corresponding sub-themes were identified (Table 2). The respondent’s quotations are represented as follows: Telangana as T, Haryana as H, Central Pollution Control Board as CPCB, State Pollution Control Board as SPCB, Centre for Science & Environment as CSE, Pharmaceutical manufacturer association of a city of Haryana as PMA-H (associations of small-medium pharmaceutical manufacturers), Organization of Pharmaceutical Producer of India as OPPI (association of multinational companies (MNCs), Bulk drug manufacturer association as BDMA, Indian Pharmaceutical Alliance as IPA (association of research based Indian companies), small-scale Indian formulation company as SSP, Medium-scale Indian formulation company as MSP, large scale Indian API company as LAPI, Multinational API manufacturing company as MNC [API], Multinational API & formulation manufacturing company as MNC [API&F].

**Table 2:** Themes and sub-themes derived through systematic thematic analysis of in-depth interviews with stakeholders

Themes	Sub-themes
Effluent treatment and disposal	(i) Practices of pharmaceutical companies (ii) Perception of stakeholders on the methodologies for effluent treatment
Common effluent treatment plants	(i) Existing standards (ii) Need for improvement of CETPs
Pharmaceutical effluents and AMR	(i) Contribution of antibiotic residues in pharmaceutical effluent towards AMR (ii) Causes other than pharmaceutical effluents contributing to AMR
Regulations for the pharmaceutical effluents containing antibiotic residues	(i) Current status (ii) Challenges

*CETP* Common Effluent Treatment Plant; *AMR* Antimicrobial Resistance

### PE treatment and disposal

This theme highlights the current PE treatment practices and perspectives of the stakeholders towards the PE treatment methodologies.

#### Practices of pharmaceutical companies

Eight pharmaceutical companies including two SMPC-H, two LAPI-H, one SMPC-T, MNC[API]-H, MNC [API]-T and MNC [API&F]-T were part of this study, and claimed to have their own effluent treatment plant (ETP) installed though respondent from one of the companies i.e., medium-scale Indian formulation company (MSP)-H mentioned that effective functionality of the ETP is subject to question due to lack of monitoring by the regulatory bodies.

Respondents from LAPI H, MNC [API&F] and MNC [API] of H and T claimed to have zero liquid discharge (ZLD) technology. ZLD involves processing the effluent through reverse osmosis (RO), to obtain clean water which can be reused. The wastewater from the RO unit (containing the pharmaceutical residues) is then processed through a multi-effect evaporator (MEE). Clean water obtained from the RO is used to form steam, which is used to run the MEE where reject water from the RO is concentrated to precipitate the dissolved salts. As per the respondent from MNC [API&F], this solid waste is then disposed off in secured landfills, or sent to the cement industry for its high calorific value as a fuel.

The effluent treatment practices of the pharmaceutical companies visited are summarized in Table 3.

**Table 3:** Practices of sewage treatment by the surveyed pharmaceutical companies

Type of pharmaceutical company	Practice of sewage treatment followed
Medium-scale Indian formulation company of Telangana	*"We are doing gel filtration, carbon filtration, double filtration, oxidation and we are checking BOD, COD these are the things we are doing in our plant and then finally we release the treated effluent into the drain."*
Multinational API manufacturing company of Telangana	*"…Now, after 7 stages—I am getting the solid out of that and the waste is liquid. Some solid may also be there. After filtration, some salt will come. That may go for land filling…secured landfill. Or if it is having calorific value, then to some cement industry. But what happens to water-based liquid—that will go to the waste treatment plant. And it's a very lengthy process. It consists of primary treatment and biological treatment, MEE, and RO plant, after that you get treated water that is used for the daily purpose..”* A respondent mentioned that ZLD exists as a rule in the Indian context however it is practically absent when analyzed in terms of practices. According to him, big companies that may follow the same rules in other countries like the U.K. but do not follow them in India
Multinational API manufacturing company of Telangana	*“We have this Zero liquid discharge plants so whatever wastewater containing any API not only antibiotic we recycle through RO systems and all. And whatever solid waste is generated will still contain some API or other things but since it is going to (dispose to) secured landfills and incineration purpose. So the spread of AMR from ZLD plant is very remote unless somebody intentionally do some mistake.”*
Small-scale Indian formulation company of Haryana	ETP installed for the treatment of effluent generated during the production of antibiotic formulations. They claimed that no liquid waste is disposed of in the sewage. The treated wastewater is used for gardening purposes and the sludge is disposed of at landfills *“that treated wastewater from ETP is used in the garden and solid waste goes to landfills”*
Medium-scale Indian formulation company of Haryana	A respondent claimed to have an ETP as per the regulations by the State Pollution Control Board but expressed concern that due to lack of active monitoring by the regulatory bodies, the regular use of the ETPs is subject to question. They informed that they have a single machine for the production of antibiotics and that after each production cycle the machine is washed but due to the absence of a sewage facility, the wastewater is stored in septic tanks. The wastewater stored in the septic tank is then used for in-house cleaning and the rest is given to the private tankers for its disposal *"There is no sewage facility so the water is put in the septic tank, the tanker people would come to collect. But they just throw it anywhere; there is no control over that. They (tanker people) throw it either in the drain or in the irrigation field"*
Large-scale Indian API manufacturing company of Haryana	*"We have put up an ETP plant and have put a flowmeter in it to check the water flow. There is this company called GEPIL. We give the sludge to them. They have a government tie-up. They (GEPIL) first check the type of the sludge. They either use it for soil filling or in the furnace. It's just like a bio-waste management system. They also take money from us."*
Large-scale Indian API manufacturing company of Haryana	*"We don't have classical ETP, we have MEE… we evaporate everything in this and the residue, salt and water is recycled."* *"That water (after treatment in the STP), we use it for irrigation. And the sludge we use for composting as manure."*
Multinational API manufacturing company of Haryana	They claimed to be using costly and energy-intensive effluent treatment technologies such as MEE, ultrafiltration, agitated thin film dryers, RO “… *we have MEE's which is very energy-intensive..We have a team of around 30 people dedicated to this..”*

*BOD* Biochemical Oxygen Demand; *COD* Chemical Oxygen Demand; *MEE* Multi-Effect Evaporator; *RO* Reverse Osmosis; *ZLD* Zero Liquid Discharge; *API* Active Pharmaceutical Ingredient; *ETP* Effluent Treatment Plant; *GEPIL* Gujarat Environment Protection & Infrastructure Ltd; *STP* Sewage Treatment Plant

#### Perception of stakeholders on the methodologies for effluent treatment

Large and multinational API companies of Haryana indicated that ZLD is unsatisfactory and the MNC [API]-H believed that ZLD/MEE is a fool proof method as only solid waste is produced which can be safely disposed in a secured landfill. The perception of various stakeholders on the methodologies for effluent treatment are detailed in Table 4.

**Table 4:** Perception of various stakeholders on the methodologies for effluent treatment

Perception on the methodologies	Stakeholders supporting the perception	Verbatims
ZLD is an unsatisfactory process	Large-scale Indian API manufacturing company of Haryana	*“They (ZLD) vaporize the water; it goes into the environment only. This is even more dangerous. This is directly going to affect the environment. -(LAPI-H)”*
	Multinational API manufacturing company of Haryana	*"If they don't measure the treated water (in ZLD) and if an antibiotic is more than the PNEC value so there are also microbes in the cooling tower so they will also get resistant.-(MNC[API]-H)"*
MEE is a fool proof method	Large-scale Indian API manufacturing company of Haryana	*"Beta-lactam antibiotics are broken based on pH, water availability and temperature at room temperature, pH is around 4.8–5, and it's an amino acid. Today after production whatever aqueous solution is coming, we are putting it to equipment called Multiple-effect evaporator virtually heating is done there, a vacuum is applied and there are 3–4 columns called distilling columns. MEE especially for API industries is recommended today.-(LAPI-H)"*
ETP is an expensive affair	Small and medium-size Indian formulation companies of Haryana & Telangana Large-scale Indian API manufacturing company of Haryana	*"They (pharmaceutical companies) do keep it (ETPs), but hardly run it. If the operational cost is 10000 for example, it is difficult for them to bear.-(SSP-H)"*
	Pharmaceutical manufacturer association	*“..the person who is able to bear the cost of ETP plants they use them and on another hand, the person who is not able to bear the cost of ETP plants, they establish the plant but they are not using them and don’t take it seriously.-(PMA-H)”*
Upgradation of technology is needed	Multinational API & formulation manufacturing company of Telangana	*"CPCB is under the impression that the simple biological treatment is sufficient which is wrong…(MNC[API&F]-T)"*
Separate industrial corridor is the solution	Medium-scale Indian formulation company of Haryana and Large-scale Indian API companies of Haryana	*"In abroad, countries have made separate zone for different industries. All alike industries in one cluster not like ours, different industries in one zone …The best solution is to have a separate industrial corridor for the pharmaceutical industry.-(H-MSP)"*
Need for specialized treatment for antibiotics	Bulk Drug Manufacturers Association	*“..see the treatment procedure depend on the type of effluent but generally they are same but certain places need specialized treatment-(BDMA)”*

*ZLD* Zero Liquid Discharge; *PNEC* Predicted No-Effect Concentration; *ETP* Effluent Treatment Plant; *H* Haryana; *LAPI* Large Scale Indian API Manufacturing Company; *MNC[API]* Multinational API Manufacturing Company; *MNC[API&F]* Multinational API & Formulation Manufacturing Company; *SSP* Small-Scale Indian Formulation Company; *PMA-H* Pharmaceutical Manufacturer Association of a City of Haryana; *T* Telangana; *MSP* Medium-Scale Indian Formulation Company; *BDMA* Bulk Drug Manufacturer Association

### Common effluent treatment plants

#### Existing standards

The official from the SPCB-T informed that small companies use common effluent treatment plant (CETPs). The effluent from the industry is brought to the CETP via tanker trucks that have online tracking system.

*“..If they dump it then that online manifest number is there, it will show.”-SPCB-T*

The official also informed that there are 4 CETPs in Hyderabad (capital city of Telangana) with one CETP having ZLD/MEE technology. Effluent with TDS greater than 5000 has to be sent to the CETP with MEE, whereas the effluent with TDS less than 5000 can be sent to either CETP. According to the official, the TDS limit is specified in the state of Telangana while other states do not have any such limits. The official also mentioned that at the ZLD facility the effluent undergoes RO and then goes to the MEE. This CETP is handled by the industries themselves with effluent being monitored by the SPCB-T and the municipal body.

*“One of the CETPs has this MEE, all the high TDS effluent of the small industries will go into that MEE.”—SPCB-T*

According to the official from the SPCB-H, the CETPs are managed by Haryana state industrial & infrastructure development corporation and the currently functioning CETPs are at two places in the State. CETPs are primarily used by small-scale units and are being updated.

#### Need for improvement of CETPs

Respondent from PMA-H suggested CETPs as the preferred solution to the effluent treatment issue. However, they argued, that there are hardly any government CETPs and the existing ones are not functioning.

The respondent from BDMA, Hyderabad also informed that small-scale industries have constrains of cost thus government should encourage their CETP usage and up-grade CETPs with adequate capacity.

The respondent from CSE mentioned that establishing an effluent treatment plant will be a challenge for the small and medium-scale pharmaceutical companies (SMPCs) hence up-graded CETPs with adequate capacity should be set up by government.

*“..It was very clearly visible during their survey that they (manufacturers) are disposing it outside, dig a hole in the ground and dispose it there, the CETP plant which was there but was not operational, and every industry's pipeline didn't reach the CETP. These few findings showed that the required waste management practice was not there.” -CSE.*

### PEs and AMR

This theme explored the perception of stakeholders towards the presence of ARs in the PE and its contribution to AMR.

#### Contribution of ARs in PE towards AMR

The respondent from CPCB informed that they are critically concerned about the issue of AMR and considered PE as a direct source of discharge of antibiotics to the environment. The respondent further highlighted that CPCB has formulated draft guidelines in which AR limits are included.

The official of the SPCB-T and SPCB-H shared the perception that ARs are absent from PE, with official from SPCB-H stating further, that no issue of AMR has been raised so far.

*“But in the outlet of the CETP we found there is no active pharmaceutical ingredient in that.”- SPCB-T.*

*“…. so if we see technically, they don’t have wastage. Only during washing of drums and floorings there will be some waste, and for that we are providing service for its treatment (at the CETPs). So, there is no particular effluent as such that will be drained out.”- SPCB-H.*

Respondent from Municipal Corporation-T mentioned that the PE have ARs but the pharmaceutical industry comes under the purview of SPCB. It was further mentioned that if an antibiotic is present in the effluent that will eventually get diluted when it comes to the sewage treatment plants. Respondents from MSP-T were aware that release of antibiotics into the environment leads to the development of AMR. Small and medium-scale formulation companies from Haryana said that possibly some amount of antibiotics is released into the environment, however, it is not a cause of concern.

*“Yes, it (antibiotics) comes out only in the washing water… And since these are all oral products, it’s fine if a little bit comes out in waste; it’s not a poison!” – MSP-H.*

The large-scale API manufacturers were aware that ARs are present in the PE, however owing to the cost, they try to extract maximum possible residues from the waste using technologies like vacuum machines and mopping.

*“Irrespective of the size of industry, be it small or medium or large – there will be wastage. Whether tablet compression or capsule filling, there will be disbursement around the machine. 0.5% or 1% wastage of antibiotic will definitely be there.”*

*– LAPI-H.*

*“… we isolated that effluent from that process and found that we are losing a lot of antibiotics in the process.” – LAPI-T.*

Respondent from PMA-H did not find presence of ARs as a cause of concern, as they are meant for human consumption.

*“Small amount of antibiotics, even if it is there, it is acceptable to our body as these antibiotics are for consumption only.” – PMA-H.*

Another respondent from PMA-H questioned the rationale behind slotting the pharmaceutical industry in the orange category with industries such as leather, paint and chemical industry when it (pharmaceutical industry) has no pollution. He further expressed that AMR is a propaganda by big companies to introduce newer molecules into the market and generate higher profits.

*“Metronidazole, norfloxacin is working very well, and then everyone talks about resistance and say we will make new antibiotics. So, resistance also comes from big companies… multinational ones, because they know that there is profit in making such drugs. If any molecule comes, hence it will give profit.” – PMA-H.*

The officials from CPCB, and respondents from LAPI-H, MSP-T, MNC [API] and MNC [API&F] of T were quite aware of the consequences of antibiotic release into the environment leading to AMR.

#### Causes other than PEs contributing to AMR

Several stakeholders stressed about other factors contributing more than PE to AMR such as municipal waste, over the counter sale of antibiotics, use in animal sector etc. as indicated by their verbatims included in the Box 1.

**Box 1:** Causes other than pharmaceutical effluents leading to AMR as mentioned by participant stakeholders

Verbatims of stakeholders regarding alternate causes as major contributors to AMR	
• *“The other thing is that it (pharmaceutical effluent) is a source of AMR but it is not only the pharmaceutical industry..It is coming from other sources also like human beings consume antibiotics and it is excreted or dispose off, it is going to the water bodies. Poultry, animals and so many other things contribute towards antibiotics in environment.-(CPCB)”* • *“There are various sources like the expired medicines are thrown into the garbage only.-(T-SPCB)”* • *"I think you will be surprised that there's a practice nowadays that some of the pharma company recycle the expired antibiotics and it is been given to the veterinarian section and misused that contributes to AMR.-(T-SPCB)”* • *“There is very high usage of antibiotics in these industries (Vet farms); if we drink milk then with this we also consume antibiotics.-(PMA-H)”* • *“we have studied and reported that the excessive use of antibiotics in animal husbandry, poultry and humans and untreated sewage have 95% contribution to AMR and only 5% comes from the diagonal places and other thing is if this is a drug mention it is localised and may have 2 or 3 industrial locations whereas all others are spread out..-(BDMA)”* • *“One reason is non-compliance of the antibiotic dosage which leads to resistance, second we are taking self-medication and third is that the antibiotics are not prescribed rightly by the doctors when it is not required it is used. Therefore, not getting the right kind of antibiotics for the right infection.-(IPA)”* • *"Hospitals contribute majorly to AMR..all the patients use antibiotics but how many hospitals have their sewage treatment plant..not even 1%. For a corporate hospital with the amount of charges and the amount of income they have; if at all they want to treat their sewage can't they afford to do this recycling? They can 100% do that…-(MNC[API]-T)"* • *"Municipal waste that is a second point source.-(MNC[API]-T)"* • *"Because antibiotic is available OTC in India so there should be legislation or some regulation from the Government of India saying that no antibiotic will be sold without prescription that will solve 60% of the purpose.-(MNC[API&F]-T)"*	

*CPCB* Central Pollution Control Board; *T* Telangana; *SPCB* State Pollution Control Board; *PMA-H* Pharmaceutical Manufacturer Association of a City of Haryana; *BDMA* Bulk Drug Manufacturer Association; *IPA* Indian Pharmaceutical Alliance; *MNC[API&F]* Multinational API & Formulation Manufacturing Company; *MNC[API]* Multinational API Manufacturing Company

### Regulations for the PEs containing antibiotic residues

#### Current status

The respondent from CPCB expressed concern about the gap in the guidelines for treatment of PE for AR and stressed that they drafted the guidelines and consultations were held with various stakeholders for their active suggestions.

The official from SPCB-T informed that the companies that generate effluent more than 25 Kilo litres per day have their ETPs. The large-scale pharmaceutical companies have installed the ZLD facility and as per the regulations they are not allowed to discharge the effluent. Whereas the official from SPCB-H informed that ZLD is mandatory for some industries but not for pharmaceutical industry in the state of Haryana. The officials from both SPCBs informed that the current regulation for the effluent does not deal with ARs.

*“We have regulations including standards for BOD, COD etc. but not for antibiotics in the effluents.-(SPCB-T)”*

The respondent from Municipal corporation, Hyderabad mentioned that the current parameters that require monitoring in the sewerage waste, established by the SPCB-T does not include antibiotics and pharmaceutical industries use their small in-built STP to treat their wastewater.

The respondents of MNC [API&F] of H and T also mentioned that there are no regulations on the treatment of PE containing ARs.

*"Regulations are not in place for antibiotic treatment, regulations have not specified any method…-(LAPI-H)"*

During the discussion with the respondents of one of the MNC [API&F]-T, it was stated that even though whatever regulations exist in the country they are not practiced.

The respondents of a MNC [API]-H mentioned that their company as a part of the AMR industry alliance is voluntarily engaged in setting up the regulatory standards.

*"There are values that have been developed for more than 140 antibiotics and these all values have been compiled by AMR industry alliance in 2018 so from that time we are quite aggressively working to achieve these values as well as to test our water and to see where we stand.-(LAPI-H)"*

One of the respondents of the Organization of Pharmaceutical Producer of India (OPPI) mentioned that member pharmaceutical companies are having a monitoring system for effluent management and internal auditing is done every quarter followed by national and global audits. It was not discussed whether the ARs are measured in the audit, most probably not as the respondents did not mention and measuring ARs is still not under any regulations.

However, the respondent from CSE highlighted that the government does not want to annoy the pharmaceutical industry which is reluctant to have regulations with levels of antibiotics in the effluent in place.

## Discussion

Our study showed that all eight pharmaceutical manufacturing units claimed to have their own functional ETPs. However, results revealed that the PE treatment and disposal practices varied. The LAPI, MNCs of Haryana and Telangana have advanced technologies including ZLD and MEE while the SMPCs have their own ETP as per the regulations. Most of the SMPCs considered effluent treatment a burden due to the cost and are often under-utilized. The lack of monitoring by the regulatory bodies was considered a major factor responsible for under-utilization of ETPs by SMPCs as well as for non-adherence to ZLD practices by MNC [API] of Telangana state. Small pharmaceutical companies use CETPs for effluent management as reported by SPCB-T and SPCB-H. It was informed by PMA-H that only a very few CETPs exist in their state and the existing ones are also not fully functional.

Though previous studies have clearly revealed the critical need for improving the PE management for removal of antibiotics to prevent development of resistant bacteria [2, 14], we found that there was a wide variation in the perception of various stakeholders about the presence of ARs in the PEs and its implications for AMR. The officials from SPCBs held a view that any effluent generated underwent treatment at CETPs and thus no active pharmaceutical ingredients is drained out. However, given the existing standards as prescribed by CPCB, effluent is not assessed for ARs. SMPCs largely view the presence of ARs in PE as inconsequential and harmless, given that antibiotics are acceptable for human consumption. Pharmaceutical association of a city of Haryana considered AMR as a propaganda to push new drugs into the market. On the other hand, most stakeholders imputed alternate causes as major attributes to AMR hence undermining the importance of PE in propagating AMR.

The representatives of MNCs were well aware of the contribution of PE to AMR and reported to be a part of AMR Industry Alliance. AMR Industry Alliance aims to provide sustainable solutions including controlling release of antibiotics during antibiotic manufacturing and establishing framework for managing pharmaceutical discharge among other measures to curb AMR [15]. In their progress report released in February 2022, AMR Industry Alliance claimed that the Alliance member sites are meeting science-based predicted no-effect concentrations (PNEC) targets for 87% of their antibiotic products in the effluents [16]. However, SMPCs in India which are significant suppliers of antibiotics domestically and to the world are not members of this association and for them removing ARs in effluent is not cost-effective.

The federal regulatory authority, CPCB recognizes PE as a direct source of discharge of antibiotics to the environment and revealed their intentions to formulate guidelines for the management of the same. WHO’s Global Leader Group on AMR has recently urged all the countries to improve management and disposal of PE containing ARs by developing and implementing regulations in its “call to action” to protect environment from antimicrobial pollution [17]. In January 2020, CPCB published draft Environment (Protection) Amendment Rules, 2019 introducing limits on ARs in the waste discharged by pharmaceutical companies, for 121 antibiotics [18, 19]. However, Indian government released the gazette notification on August 6, 2021, as amendment to Environment (Protection) rules, 1986 but did not include the limits for ARs in PE [12]_._ Our study points out operational costs as a major concern in the treatment of PE by the small-middle scale pharmaceutical companies. The reason for exclusion of limits for ARs in PE could perhaps be because stringent regulations would result in losses for small-medium scale pharmaceutical companies, thus raising prices and impacting access to antibiotics [19]. However, due to the potential AMR implications there should be efforts to reduce ARs in the effluent. More importantly, the draft bill demonstrated the federal government’s intent to regulate ARs in PE, the MNCs are voluntarily engaged, the SMPCs are willing to pay for services from government operated CETPs and the states await central regulations. The state of Telangana has recently explored public private partnership model by outsourcing the maintenance and operations of a newly planned CETP to a private sector entity, with mandates for real-time monitoring of effluents [20, 21]. Therefore, government funded CETPs, government operated chargeable CETPs and public private partnership, are potential ways of regulating ARs in PEs in India.

Strengths of the study is involvement of important stakeholders including regulators for pharmaceutical effluents, various pharmaceutical industry associations and pharmaceutical manufacturing sector of different capacity (small, medium and large manufacturing units). However, there are some limitations to the study. We included regulators and pharmaceutical manufacturing units only from two states while antibiotic manufacturing units are present in several states. The other states could have different practices and TDS limits may vary for PE treatment. Therefore, we need to be cautious in generalizing the study findings to other states in India.

## Conclusion

Addressing the presence of antibiotics in pharmaceutical effluents, is critical to curbing the spread of AMR. However, India is faced with challenges such as lack of awareness, economic feasibility and lack of regulation. The several implications that arise from this study, could aid in systematic approach in policy making and capacity-building in dealing with the ARs in PE to combat AMR in countries like India. Firstly, the government should set up regulations with clear limits for each antibiotic in the PEs and ensure strict implementation and monitoring. Secondly, standardized CETPs with effluent testing laboratories for management of ARs in the PE should be set up by the government itself for the SMPCs as these companies are willing to pay for the same but could not afford individual set-up for effluent management. Thirdly, large and multinational pharmaceutical industries should improvise their effluent management system for sustainable antibiotic production and strictly follow the limits set up for the ARs in the PE. Fourthly, the public private partnership between government and pharmaceutical associations could also work for maintaining the CETP and monitoring ARs levels in the treated effluents. Finally, the awareness programs should be carried out with involvement of all major stakeholders including policy makers, manufacturers, civil society as well as academia to improve knowledge which would ultimately influence the practices to minimize the contribution of ARs in PEs to AMR.

## Data Availability

All the data will be made available after receiving the request.

## Abbreviations

AMR: Antimicrobial Resistance
API: Active Pharmaceutical Ingredient
AR: Antibiotic Residue
BDMA: Bulk Drug Manufacturer Association
BOD: Biochemical Oxygen Demand
CETP: Common Effluent Treatment Plant
COD: Chemical Oxygen Demand
CPCB: Central Pollution Control Board
CSE: Centre for Science & Environment
ETP: Effluent Treatment Plant
FDP: Finished Dose Product
IPA: Indian Pharmaceutical Alliance
LAPI-H: Large-Scale Indian API Company-Haryana
LAPI-T: Large-Scale Indian API Company-Telangana
MEE: Multi-Effect Evaporator
MNC [API&F]-T: Multinational API and Formulation Manufacturing Company-Telangana
MNC [API]-H: Multinational API Manufacturing Company-Haryana
MNC [API]-T: Multinational API Manufacturing Company-Telangana
MNC: Multinational Company
MSP-H: Medium-scale Formulation Pharmaceutical Company-Haryana
MSP-T: Medium-scale Formulation Pharmaceutical Company-Telangana
OPPI: Organization of Pharmaceutical Producer of India
PE: Pharmaceutical Effluent
PFI: Pharmaceutical Formulation Intermediates
PMA-H: Pharmaceutical Manufacturer Association-Haryana
PNEC: Predicted No-Effect Concentrations
RO: Reverse Osmosis
SMPC: Small and Medium-scale Pharmaceutical Company
SPCB-H: State Pollution Control Board-Haryana
SPCB-T: State Pollution Control Board-Telangana
SSP-H: Small-Scale Formulation Pharmaceutical Company-Haryana
TDS: Total Suspended Solids
ZLD: Zero Liquid Discharge
